# Impact of the “Stoptober” Smoking Cessation Campaign in England From 2012 to 2017: A Quasiexperimental Repeat Cross-Sectional Study

**DOI:** 10.1093/ntr/ntz108

**Published:** 2019-06-27

**Authors:** Mirte A G Kuipers, Robert West, Emma V Beard, Jamie Brown

**Affiliations:** 1 Department of Public Health, Amsterdam UMC, University of Amsterdam, Amsterdam, The Netherlands; 2 Department of Behavioural Science and Health, University College London, London, UK; 3 Research Department of Clinical, Educational and Health Psychology, University College London, London, UK

## Abstract

**Introduction:**

Since 2012, England has an annual “Stoptober” campaign for collective smoking cessation. Our aim was to assess (1) overall impact of the Stoptober campaign on quit attempts over its first 6 years, (2) consistency of impact over the campaign years, and (3) the role of the campaign budget.

**Methods:**

We used data of 51 399 adult smokers and ex-smokers in 132 repeat cross-sectional monthly surveys in England, 2007–2017. In a quasiexperimental design, adjusted logistic regression analyses compared past-month quit attempt rate between (1) October and other months in the year, between 2007–2011 and 2012–2017; (2) October and other months, across years 2012–2017; and (3) October and other months, between high-budget (2012–2015) and low-budget Stoptober campaigns (2016–2017). Bayes factors (BF) differentiated insensitive data and absence of an effect.

**Results:**

(1) In 2012–2017, quit attempts were more prevalent in October versus other months (odds ratio [OR]: 1.24, 95% confidence interval [CI]: 1.00 to 1.53), whereas similar in 2007–2011 (OR: 0.95, 95% CI: 0.76 to 1.18; BF = 0.2); data were somewhat insensitive but supported this difference (OR: 1.30, 95% CI: 0.97 to 1.75; BF = 2.1). (2) In 2012–2017, quit attempt prevalence ranged from 3.1% to 8.5% in October and 5.0% to 7.3% in other months. The difference between October and other months was large in 2012 (absolute unadjusted difference of 3.3%; OR: 1.92, 95% CI: 1.23 to 2.98) and 2015 (3.1%; OR: 1.84, 95% CI: 1.14 to 2.95), but small in 2013–2014 and 2016–2017 (0.36 < BF < 1.02). (3) Data were somewhat insensitive but supported interaction with campaign budget (OR: 1.50, 95% CI: 0.92 to 2.44; BF = 2.2).

**Discussion:**

In 2012–2017, there appears to have been an increase in past-month quit attempts during October in England. The increase was inconsistent across Stoptober campaigns and appears to have been greater when the campaign budget was higher.

**Implications:**

Over the first 6 years of Stoptober campaigns, there appears to have been an overall increase in past-month quit attempts during October in England, and the data imply that a sufficiently high budget contributes to greater impact of the Stoptober campaign. These findings encourage the further spread of the Stoptober campaign to other countries. Future research should clarify how increased quit attempts as a consequence of Stoptober translate into quit success and which of Stoptober’s ingredients were most important in increasing quit attempts, especially among vulnerable groups.

## Introduction

Stoptober is a smoking cessation campaign that encourages smokers to abstain from smoking for 28 days during the month of October.^1^ It was first implemented in England in 2012, and versions of it have since been adopted in other countries, such as New Zealand, the Netherlands, and France, following a positive evaluation of the first campaign.^1^ The evaluation of the 2012 campaign estimated that Stoptober increased the odds of making a quit attempts in October by 80%, generated an additional 350 000 quit attempts, and would have gone on to save 10 400 discounted life years at less than £415 per discounted life year.^1^ In the Netherlands, 2014–2016 Stoptober campaigns were associated with an increase in online searches for smoking cessation,^2^ and among participants, smoking-outcomes improved.^3^ It is important to understand the impact of Stoptober over a longer period of time, to inform future Stoptober campaigns, as well as other interventions.

Stoptober was designed to create a national collective effort to abstain for 28 days in October as a stepping stone to permanent cessation. The campaign involves setting a 28-day smoke-free target and use of positive messages conveyed through a combination of traditional and digital media including TV, press, radio and online advertisements, public relations, Facebook, and Twitter. Smokers who sign up gain access to a range of quitting tools.^1^

Following the positive evaluation of the first event in 2012,^1^ the campaign has now run for 6 consecutive years and is embedded within the tobacco control landscape of England. Public Health England, who run the campaign, have internally performed annual marketing evaluations, but effectiveness of campaigns has not been evaluated against a population-wide sample since 2012. There is a need to establish whether the impact associated with the 2012 campaign has been sustained year on year. The impact may have sustained or even grown because the campaign gained momentum from increased recognition. Evaluation of No Smoking Day in England has shown continued impact over a number of years.^4^ Alternatively, the impact may have declined with time as the target group became inured or desensitized to the messaging with the repetitions of the campaign.^5^ Such a “wear-out” effect has also been demonstrated for cigarette health warning labels when messages are not regularly updated.^6^

Independently, the campaign budget for Stoptober may play an important role in the impact over time. The budget was high in the first years but has been substantially reduced in recent years. The impact of such a decline in expenditure may reduce campaign effectiveness, given that higher tobacco control mass media campaign expenditures have been associated with higher quit success rates.^7^

This article aimed to assess the overall impact of the Stoptober campaign over its first 6 years in England. In England, the smoking prevalence in the general adult population has decreased from 24.2% in 2007 to 20.0% in 2012 and 17.2% in 2017. The proportion of smokers reporting to have tried to quit over the past year has however decreased from 42.2% in 2007 to 34.4% in 2012 and 30.2% in 2017 (www.smokinginengland.info). In this study, we specifically aimed to answer the following research questions:

1. What was the difference in quit attempt rates in October versus other months of the year in 2012–2017 (after Stoptober was implemented) versus 2007–2011 (before Stoptober was implemented)?
2. Did the difference in quit attempt rates between October and other months change over the years 2012 to 2017?
3. Did the difference in quit attempt rates between October and other months differ between Stoptober campaigns with a high budget and Stoptober campaigns with a low budget?

## Methods

### Study Population

We used monthly data of representative samples of the English adult population from the Smoking Toolkit Study. Since November 2006, Smoking Toolkit Study selects a new sample each month of approximately 1750 adults aged at least 16 years using a form of random location sampling. After stratification by geo-demographic classification of the population, output areas containing approximately 300 households are allocated randomly to interviewers who conduct interviews in those areas until a pre‐specified quota tailored to the area is reached. The interview is face to face and computer assisted with one member of a household by a trained interviewer. Response rates cannot be calculated because of the lack of a definitive gross sample: all units fulfilling the criteria of a given quota within each area are interchangeable. Ethical approval was obtained from the University College London Ethics Committee (ID 0498/001). Full details of the Smoking Toolkit Study have been described elsewhere.^8^

This study used 11 years of monthly data, collected between January 2007 and December 2017 (*N* = 233 547). We included respondents who reported that they smoked in the past year (*n* = 53 717) and had not stopped smoking more than 1 month before the survey (*n* = 51 531). We excluded respondents with missing values for any variable described later (86 on age, 44 on quit attempts, and 3 on gender) leading to a final sample size of 51 399, with 4348 individuals interviewed in October months.

### Study Design

The Stoptober campaign acted as a “natural experiment,” which was evaluated in a quasiexperimental repeat cross-sectional study design, extending methodology used in the first year evaluation of Stoptober.^1^ We expected a higher quit attempt prevalence in October than other months in the year, in the years in which the Stoptober campaign ran. To attribute this difference to Stoptober, this difference should be larger in Stoptober years 2012–2017 than in pre-Stoptober years 2007–2011.

Stoptober encourages smokers to abstain from smoking for 28 days during October. Behavior change techniques underpinned by key psychological principles of social contagion theory, SMART goals, and PRIME theory^1^ were applied in the two key elements of the campaign: (1) the start of a national movement, in which smokers collectively quit smoking at the same time, through mass media messaging and (2) providing a wide range of support tools to achieve the SMART goal to quit for 28 days, while broadcasting the positive message that any smoker would be five times more likely to succeed permanently when realizing this goal.

### Measures

#### Dependent Variable

Making a quit attempt was used as the outcome variable. Individuals who made at least one quit attempt in past 12 months (“How many serious attempts to stop smoking have you made in the last 12 months?” ≥ 1) and started this attempt up to 1 month ago (“How long ago did your most recent serious quit attempt start?”≤1 month) were coded 1, all others were coded 0.

#### Independent Variables

Three Stoptober-related variables were measured. We distinguished the month October from all other months of the year (October = 1, Other months = 0). The period before the launch of the Stoptober campaign (2007–2011) was coded 0, and the year in which the campaign ran (2012–2017) was coded 1. The budget used for the Stoptober campaign in each year in 2012–2017 was measured in m£ and was divided into high (2012–2015) and low (2016–2017) budget. Information was provided by Public Health England.

Trend over years was measured by coding months throughout the study period, ranging 1–132. Trend within years was captured by coding months within the year ranging 1–12. Years within the Stoptober-period (2012–2017) were distinguished (range 1–6).

Sociodemographic variables of gender (female = 0, male = 1), age (continuous variable, divided by 10 to reflect 10-year increases in age), and social grade were included as confounders. Social grade distinguished lower (manual occupation National Readership Survey (NRS) social grades C2, D, and E) and higher (non-manual occupation NRS social grades AB and C1).

Three variables at the country-level were included to control for other developments that occurred over time that may have affected quit attempt prevalence. First, a variable reflected the course of tobacco control policies in England. The value increased by one unit with the introduction of each new policy in the study period (January 2007–December 2017) and was assigned to the month of when the policy was implemented. Over time, the variable values ranged from 1 to 9, with one point added when the following policies were implemented: (1) July 2007: smoking ban in enclosed premises and public vehicles. (Hotel rooms, prisons, and nursing homes excluded); (2) October 2009: pictorial warnings on cigarette packs; (3) October 2010: pictorial warnings on all tobacco products; (4) October 2011: ban on sale of tobacco from vending machines; (5) October 2013: ban advertising at the point of sale; (6) April 2013: ban on displaying cigarette packs in large shops; (7) April 2015: ban on displaying cigarette packs in small shops; and (8 + 9) May 2016: start transition to standardized packs, + TPD part 1: larger pictorial health warnings, product content changes.

Second, we created a variable that reflected the increase in excise tax from each March budget announcement onward, in the % increase above inflation compared with the previous year. In case of disproportionate tax on roll-your-own (RYO) tobacco, the RYO tax increase weights for 1/3 (similar to the level of use). Tax increase levels were derived from data of Action on Smoking and Health: 2001–2008: in line with inflation (0%), 2009: 2%, 2010: 1%, 2011: 2%, and 10% on hand-rolled (4.6%), 2012: 5%, 2013: 2%, 2014: 2%, 2015: 2%, 2016: 2%, and 5% on hand-rolled (3%), 2017: 2%.

Third, a variable expressed the expenditure on mass media campaigns other than Stoptober in £millions per month. Expenditure was zero in months during which no campaign ran. Budget information was provided by Public Health England.

### Statistical Analyses

The analysis plan was registered on the Open Science Framework before data analysis (https://osf.io/ab3vu/). For all analyses, Stata, version 15.1 was used.

First, we obtained descriptive statistics for the sociodemographics of the study population and quit attempt prevalence in October months and other months, in the Stoptober period (2012–2017) and pre-Stoptober period (2007–2011).

Second, the impact of Stoptober was investigated. A logistic regression was performed with quit attempts as the outcome and October versus other months and Stoptober-period versus pre-Stoptober period as independent variables. Model 1 controlled for time variables (trend over years and within year) and sociodemographics (age, gender, social grade). Model 2 additionally controlled for country-level variables for tobacco control policies, tax increases, and mass media campaign expenditure. In Model 3, we added an interaction term for October × Stoptober to test whether the difference in quit attempts between October months and other months was larger in the Stoptober period (2012–2017) than in the pre-Stoptober period (2007–2011).

Third, we assessed the consistency of the impact of Stoptober over the years 2012–2017. Logistic regression of quit attempts on to the year of the Stoptober campaign, October versus other months, and interaction between year × October. The analysis was controlled for all covariates.

Fourth, the role of the Stoptober campaign budget was examined with a logistic regression model on data from 2012 to 2017. The model included the campaign budget and interaction between October × campaign budget and adjusted for all covariates. We derived the odds of quit attempt in October versus other months of the year, within each year, and the difference in odds of quitting in October instead of other months, between 2012 and each consecutive year.

Bayes factors (BF) were calculated, as a means of differentiating insensitive data and evidence supporting the absence of an effect. We used H1 as a half-normal distribution with SD of the natural log of the odds ratio (ie, the regression coefficients) as reported by Brown *et al*.,^9^ for which the plausible effect size was defined as odds ratios found in Brown *et al*..^1^ We used the online calculator: http://www.lifesci.sussex.ac.uk/home/Zoltan_Dienes/inference/bayes_factor.swf.

All regression analyses were performed on weighted data using the rim (marginal) weighting technique to match English census data on age, sex, and socioeconomic group. In two sensitivity analyses, the main analysis on the impact of Stoptober was repeated on unweighted data and in a model that was not adjusted for time variables. Results presented in Supplementary Table 1 show that the results did not substantially diverge from the main analysis.

## Results

Supplementary Table 2 presents a description of the study population. The mean age was 42.5 years, approximately half of respondents were female, and almost two-thirds were of lower social grades. Age and gender were similar between October and other months, and between the Stoptober period and pre-Stoptober period, but the proportion of individuals from lower social grades was higher in earlier years.

**Figure 1.**
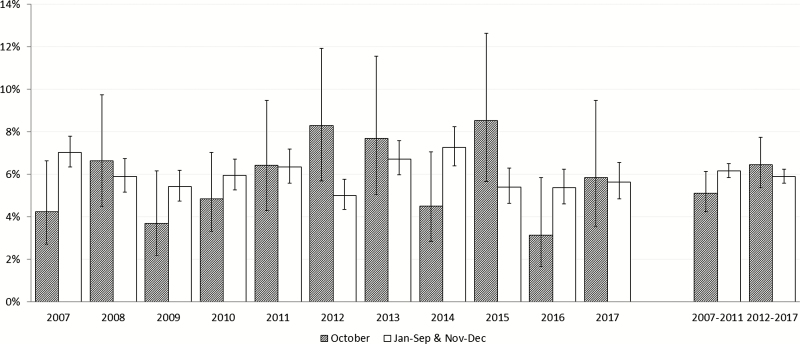
Quit attempt (% quit attempts that started up to a month before the survey interview) in October and other months of the same year, in 2007–2017.

Figure 1 shows that the quit attempt prevalence varied between the years. In the Stoptober period (2012–2017), it ranged from 3.1% to 8.5% in October months and from 5.0% to 7.3% in other months of the year. Although in the pre-Stoptober period the quit attempt prevalence was lower in October (5.1%) than in other months of the year (6.2%), it was higher in the Stoptober period (6.5% in October vs. 5.9% in other months). However, 95% confidence intervals (CIs) for these unadjusted figures overlapped.

Supplementary Figure 1 presents trends in country-level variables. Expenditure on mass media campaigns other than Stoptober fluctuated over time, with a substantial hiatus in 2010–2011. Taxes increased more in 2011 and 2012 than in other years. The Stoptober campaign budget was substantially higher in 2012–2015 than in 2016–2017.

Table 1 presents the logistic regression results on odds of making a quit attempt in October versus other months, and in the Stoptober period versus pre-Stoptober period. Models 1 and 2 demonstrated that over the 11 years of the study, there was no difference in quit attempts between October and other months of the year (Model 2 odds ratio [OR]: 1.09, 95% CI: 0.93 to 1.27). Models 1 and 2 also show that there was no difference in overall quit attempts between the Stoptober years and pre-Stoptober years (Model 2 OR: 1.16, 95 CI: 0.97 to 1.38). In Model 3, the interaction term between October and Stoptober was included. This means that the OR for October can be interpreted as the difference between October and other months of the year within the pre-Stoptober period. This difference was not significant (OR: 0.95, 95% CI: 0.76 to 1.18, BF: 0.19). We can derive the OR for October within the Stoptober period, which showed higher odds of quitting in October versus other months of the year (OR: 1.24, 95% CI: 1.00 to 1.53, BF: 3.03). The interaction was not significant but supported there being an overall change (OR: 1.30, 95% CI: 0.97 to 1.75, BF: 2.08).

**Table 1.** Logistic regression models for the weighted odds of having made a quit attempt in the last month. *n* = 51 399

	Odds ratio (95% confidence interval)		
	Model 1	Model 2	Model 3
October^a^	1.07 (0.92 to 1.25)	1.09 (0.93 to 1.27)	0.95 (0.76 to 1.18)^c^
Stoptober^b^	1.09 (0.93 to 1.28)	1.16 (0.97 to 1.38)	1.12 (0.94 to 1.34)^d^
Interaction October × Stoptober	—	—	1.30 (0.97 to 1.75)^e^
Time			
Month within year, per month increase	0.97 (0.96 to 0.99)	0.97 (0.96 to 0.98)	0.97 (0.96 to 0.98)
Month of study, per year increase	0.98 (0.96 to 0.99)	0.96 (0.89 to 1.04)	0.97 (0.88 to 1.03)
Sociodemographics			
Age, per 10 years increase	0.89 (0.87 to 0.91)	0.89 (0.87 to 0.91)	0.89 (0.87 to 0.91)
Male gender vs female	0.96 (0.89 to 1.04)	0.96 (0.87 to 1.04)	0.96 (0.89 to 1.04)
Higher social grade vs lower	0.96 (0.91 to 1.07)	0.99 (0.91 to 1.07)	0.99 (0.91 to 1.07)
Country-level covariates			
Tobacco control policies, per 1 point increase		1.01 (0.93 to 1.11)	1.02 (0.94 to 1.12)
Tax increases, per 1% increase above inflation		0.98 (0.94 to 1.01)	0.98 (0.95 to 1.01)
Mass media campaign expenditure, per 1£m increase		0.91 (0.85 to 0.98)	0.91 (0.85 to 0.98)

^a^October coded as 1 = October versus 0 = other months of the year.

^b^Stoptober coded as 1 = Stoptober period 2012–2017 versus 0 = pre-Stoptober period 2007–2011.

^c^Odds ratio (OR) represents difference between October and other months of the year within the pre-Stoptober period.

^d^OR represents difference between Stoptober period and pre-Stoptober period within months of the year other than October.

^d^OR represents difference in odds of quitting in October instead of other months, between Stoptober period and pre-Stoptober period.

Table 2 assessed the consistency in the differences between October and other months of the year over the Stoptober campaigns. The upper part of the table shows the results within years, and the lower part compares these results relative to 2012. In 2012 and 2015, the odds of making a quit attempt were higher in October than in other months of the year (OR 2012: 1.92, 95% CI: 1.23 to 2.98; OR 2015: 1.84, 95% CI: 1.14 to 2.95). In 2014 and 2016, this association was significantly weaker than in 2012 (OR 2014: 0.34, 95% CI: 0.38 to 0.66; OR 2016: 0.35, 95% CI: 0.15 to 0.78), and in these years the BF tended to favor the hypothesis of no effect.

**Table 2.** Logistic regression models for the weighted odds of having made a quit attempt in the last month, comparison of years within Stoptober period (2012–2017). *n* = 26 611

	Odds ratio (95% confidence interval)^a^	*p*-value	Bayes factor
October^b^			
2012	1.92 (1.23 to 2.98)	.004	23.73
2013	1.23 (0.76 to 1.97)	.399	1.02
2014	0.66 (0.40 to 1.09)	.106	0.23
2015	1.84 (1.14 to 2.95)	.012	10.49
2016	0.67 (0.34 to 1.32)	.244	0.36
2017	1.09 (0.63 to 1.91)	.752	0.71
October × year^c^			
2012	Ref		
2013	0.64 (0.34 to 1.21)	.172	0.23
2014	0.34 (0.38 to 0.66)	.001	0.13
2015	0.96 (0.51 to 1.82)	.899	0.45
2016	0.35 (0.15 to 0.78)	.011	0.19
2017	0.57 (0.28 to 1.15)	.117	0.23

^a^Adjusted for month of the year, age, gender, social grade, cumulative tobacco control policy score, tax increases, mass media campaign expenditure.

^b^October coded as 1 = October versus 0 = other months of the year. Odds ratios (Ors) represent odds of quit attempt in October versus other months of the year, within each year.

^c^ORs represent difference in odds of quitting in October instead of other months, between 2012 and each consecutive year.

Table 3 examines the role of the Stoptober campaign budget. In years with high campaign spending the odds of making a quit attempt were higher in October than in other months (OR: 1.35, 95% CI: 1.06 to 1.73), whereas this association was not found in years with low campaign spending (OR: 0.90, 95% CI: 0.59 to 1.39, BF: 0.45). The interaction was not significant but weakly supported there being an overall difference (OR: 1.50, 95% CI: 0.92 to 2.44, BF: 2.21).

**Table 3.** Logistic regression for the weighted odds of having made a quit attempt in October compared with other months, comparison of high budget and low budget Stoptober campaigns within Stoptober period (2012–2017). *n* = 26 611

	Odds ratio (95% confidence interval)^a^	*p*-value	Bayes factor
October^b^			
Low	0.90 (0.59 to 1.39)	.643	0.45
High	1.35 (1.06 to 1.73)	.017	5.80
Stoptober budget × October^c^			
Low	Ref		
High	1.50 (0.92 to 2.44)	.106	2.21

^a^Adjusted for month of the year, month of the study, age, gender, social grade, cumulative tobacco control policy score, tax increases, mass media campaign expenditure.

^b^October coded as 1 = October versus 0 = other months of the year, budget coded as high = 2012–2015 versus low = 2016–2017. Odds ratios (Ors) represent odds of quit attempt in October versus other months of the year, within high budget and low budget Stoptober campaigns.

^c^OR represents difference in odds of quit attempt in October versus other months of the year, between high budget and low budget Stoptober campaigns.

## Discussion

### Key Findings

The data were somewhat insensitive but supported there being an overall change between pre-2012 and 2012 onward in the difference between quit attempts in October and other months: in 2012–2017, quit attempts were more prevalent in October versus other months, whereas the prevalence was similar in 2007–2011. The difference in attempts between October and other months was large in 2012 and 2015, but similar in 2013–2014 and 2016–2017. We found weak support for the difference between attempts in October and other months being larger in years with high Stoptober campaign budgets than years with low campaign budgets, although the data were insensitive.

### Strengths and Limitations

This study used a quasiexperimental design to assess the real-world impact of the Stoptober campaign over 6 consecutive years. We consider it likely that associations found were causal, as we thoroughly adjusted for confounding factors and did not find plausible other changes or events influencing quit attempt rates occurring around October in 2012–2017, but not in 2007–2011. A further strength of the study is the use of a nationally representative ongoing survey, yielding comparable data over the entire 11-year study period. Self-reporting of quitting behavior was obtained without reference to Stoptober, which lowered the risk of reporting bias.

There are however some limitations that need to be taken into account when interpreting the findings. As all data on quit attempts were self-reported, past-month quitting may be misclassified by some respondents because of recall bias and/or desirability bias. Although we expect some degree of recall and desirability bias in all months of the survey, more respondents may have reported quitting in Stoptober months, if they felt social pressure to participate in the mass quit attempt. We cannot establish whether this potential side effect of Stoptober exists and if it would lead to overestimation of the actual impact on quit attempts. Misclassification may have also occurred because the exact timing of the interview could not be taken into account. Respondents reported whether they had started their quit attempt no longer than a month ago, and respondents who were interviewed at the beginning of a month may have reported on the previous month. It is unlikely that this has occurred more in October than in November and therefore has not resulted in an overestimation of the impact of Stoptober. A second limitation is that we did not take the level of campaign exposure into account and the elements of the campaign to which individuals were exposed. We can therefore only draw an overall conclusion on effectiveness of the campaign at the population level, but not on specific campaign elements or the optimal exposure level for individuals. A third limitation is that we were only able to characterize differences between campaigns in terms of the budget. Although the overall thrust of the campaign and key principles remained constant, it is likely there were other differences in the campaigns across years, which may have affected the effectiveness.

### Comparison With Previous Studies

This study builds on the evaluation of the 2012 Stoptober campaign, which used the same data source up to 2012.^1^ This analysis showed that additional adjustment for time-dependent variables did not diminish the previously demonstrated impact. The impact on quit attempts is also in line with studies on other mass media campaigns aimed at promoting quitting.^10,11^ A study conducted between 2005 and 2010 in the United Kingdom found that positive campaign messages were more effective at increasing quitline calls (ie, an indicator of quit attempts) than negative messages.^12^ In the United States the EX smoking cessation mass media campaign has been successful in increasing quit attempts as well.^13^ EX is different from Stoptober in that it does not set a mass-quitting date, but similar in its empathetic and positive approach.

### Interpretation of the Findings

Stoptober was found to increase the prevalence of quit attempts undertaken in October. Although we cannot distinguish which campaign ingredients were most important, the behavior change techniques that were based on psychological theory appear successful. Stoptober’s use of social support as a behavior change technique to quit smoking is not a new concept. In stop smoking services, the provision of social support has been associated with improved short-term abstinence and higher quit success rates.^14–16^ In 2009, 37% of stop smoking services applied social support techniques,^15^ and recognition of the importance of social support in stop smoking services has since increased.^16^ This study suggests that the Stoptober campaign can scale up social support to the wider population and that it may further support the effectiveness of stop smoking services.

In our data, the past-month quit attempt prevalence was somewhat higher across the full year in 2012–2017 than pre-2012, although not statistically significant. This suggests that at least part of the quit attempts undertaken during the Stoptober campaign are additional quit attempts that would not have been undertaken in a different month of the year in absence of the campaign. Stoptober therefore may have successfully encouraged smokers who would not have otherwise undertaken a quit attempt in the same year.

We found that higher campaign budgets were associated with a larger increases in quit attempts, but in a previous English study, the association between overall mass media expenditure levels and changes in concurrent quit attempts could not be demonstrated.^7^ In the Stoptober campaign, the budget did determine not only the level of exposure to ads through various mass media channels but also the development and distribution of support tools. Although a higher budget may improve campaign content, reach and provided support, higher budgets did not consistently lead to high impact. We found a lack effect in 2014 and 2016, whereas there was a considerable difference in campaign budget between the 2 years. This variation in impact over the years speaks against a general “wear-out” effect or an improvement of effectiveness over time. The results suggest that other factors must be at play.

In the longer term, Stoptober would only have been effective in lowering smoking prevalence if quit attempts are at least equally successful as attempts made in the rest of the year. Results from a longitudinal study from the Netherlands among Stoptober participants showed that about half of smokers had remained quit after 3 months.^3^ The Smoking Toolkit Study data showed that both the annual quit rate (% former smokers among ever-smokers) and the quit success rate among smokers who tried to stop smoking over the past year showed a stronger increase in 2012–2017 than in 2007–2011. Although no direct evidence, it supports that Stoptober did not only increase quit attempts but also sustained quitting. However, in a qualitative study in the Netherlands, Stoptober participants expressed the need for continued support after the campaign to sustain smoking cessation in the long term.^17^

### Implications

The early positive results on the effectiveness of the Stoptober campaign encouraged the further spread of the campaign to other countries. It may also have implications for other health (risk) behaviors. The UK campaign Dry January was launched a year after Stoptober and challenges people to stop drinking alcohol for a month. Many of the same principles of positive messaging and social support were applied. Studies on alcohol use in and around the month of January showed promising results in the moderation of alcohol use in January,^18,19^ although quasiexperimental population-level studies are lacking. A fundamental difference between Stoptober and Dry January, and comparable initiatives, is that the goal rarely is to quit drinking permanently.

The current studies leave a number of questions unanswered that require further study. We were unable to measure the population impact of Stoptober on quit success as the timing of the attempt and the period of which it would last could not be measured in sufficient detail. Furthermore, this study did not unravel which ingredients of the campaign were most important in increasing quit attempts, and which elements can be added or adapted to reach more vulnerable groups, and increase successful completion rate.

## Conclusions

Over the first 6 years of Stoptober campaigns, there appears to have been an overall increase in past month quit attempts during October in England. The associated increase was inconsistent across campaigns and findings imply that a sufficiently high budget needs to be secured for future campaigns.

## Funding

The Smoking Toolkit Study is currently primarily funded by Cancer Research UK (C1417/A14135; C36048/A11654; C44576/A19501) and has previously also been funded by Pfizer, GSK, and the Department of Health. MAGK is funded by an EC Horizon 2020 grant (SILNE-R, grant agreement no. 635056); RW is funded by Cancer Research UK (C1417/A14135); EB is funded by a fellowship from the NIHR SPHR (SPHR-SWP-ALC-WP5) and Cancer Research UK (CRUK) also provide support (C1417/A14135); JB’s post is funded by a fellowship from the Society for the Study of Addiction and CRUK also provide support (C1417/A14135).

## Declaration of Interests

RW undertakes consultancy and research for and receives travel funds and hospitality from manufacturers of smoking cessation medications but does not and will not take funds from e-cigarettes manufacturers or the tobacco industry. RW is honorary co-director of the National Centre for Smoking Cessation and Training (NCSCT). RW is a Trustee of the stop-smoking charity, QUIT. EB and JB have received unrestricted research funding from Pfizer. EB and JB are funded by CRUK. EB is also funded by National Institute for Health Research (NIHR), School for Public Health Research (SPHR). MAGK has no interests to declare.

## Supplementary Material

ntz108_Suppl_Supplementary_Material
