# Supplementary material for: Impact of the “Stoptober” Smoking Cessation Campaign in England From 2012 to 2017: A Quasiexperimental Repeat Cross-Sectional Study
Source: Nicotine Tob Res. 2019 Jun 27;22(9):1453–9. doi: 10.1093/ntr/ntz108 (PMC7443602; doi:10.1093/ntr/ntz108)
Supplement: ntz108_Suppl_Supplementary_Material [file ntz108_suppl_supplementary_material.pdf]

## SUPPLEMENT

**Supplementary Table 1:** Sensitivity analyses of main associations, on unweighted data, and on weighted data without adjustment for time variables. N=51,399.

| | Odds ratio (95% confidence interval) | |
|---|---|---|
| | **Without weights[a]** | **Without adjustment for time[b]** |
| OR for October[c] within Stoptober period (2012-2017) | 1.24 (1.02-1.50) | 1.10 (0.89-1.35) |
| OR for October[c] within pre-Stoptober period (2007-2011) | 1.02 (0.84-1.24) | 0.84 (0.69-1.04) |
| Stoptober period compared with pre-Stoptober period (i.e. interaction October* Stoptober)[d] | 1.22 (0.93-1.59) | 1.30 (0.97-1.74) |

[a] Adjusted for month of the year, month of the study, age, gender, social grade, cumulative tobacco control policy score, tax increases, mass media campaign expenditure.
[b] Adjusted for age, gender, social grade, cumulative tobacco control policy score, tax increases, mass media campaign expenditure.
[c] October coded as 1=October vs 0=other months of the year.
[d] ORs represent difference in odds of quitting in October instead of other months, between Stoptober period and pre-Stoptober period.

**Supplementary Table 2:** Unweighted description of the study population.

| | **Total population** | **Stoptober period (2012-2017)** | | **Pre-Stoptober period (2007-2011)** | |
|---|---|---|---|---|---|
| | | **October** | **Jan–Sept & Nov–Dec** | **October** | **Jan–Sept & Nov–Dec** |
| N respondents | 51,399 | 1,973 | 22,815 | 2,375 | 24,236 |
| Mean age (95%CI) | 42·5 (42·4-42·7) | 43·0 (42·2-43·7) | 42·7 (42·4-42·9) | 42·7 (42·0-43·4) | 42·3 (42·1-42·6) |
| % Female (95%CI) | 48·7 (48·3-49·2) | 47·5 (45·3-49·7) | 46·6 (46·0-47·3) | 50·8 (48·8-52·8) | 50·7 (50·0-51·3) |
| % Lower social grade (95%CI) [a] | 65·4 (64·9-65·8) | 63·7 (61·5-65·8) | 63·2 (62·6-63·8) | 69·9 (68·1-71·7) | 67·0 (66·5-67·7) |

[a] Social grades divided in lower: manual occupation (National Readership Survey (NRS) social grades C2, D and E) and higher: non-manual occupation (NRS social grades AB and C1).

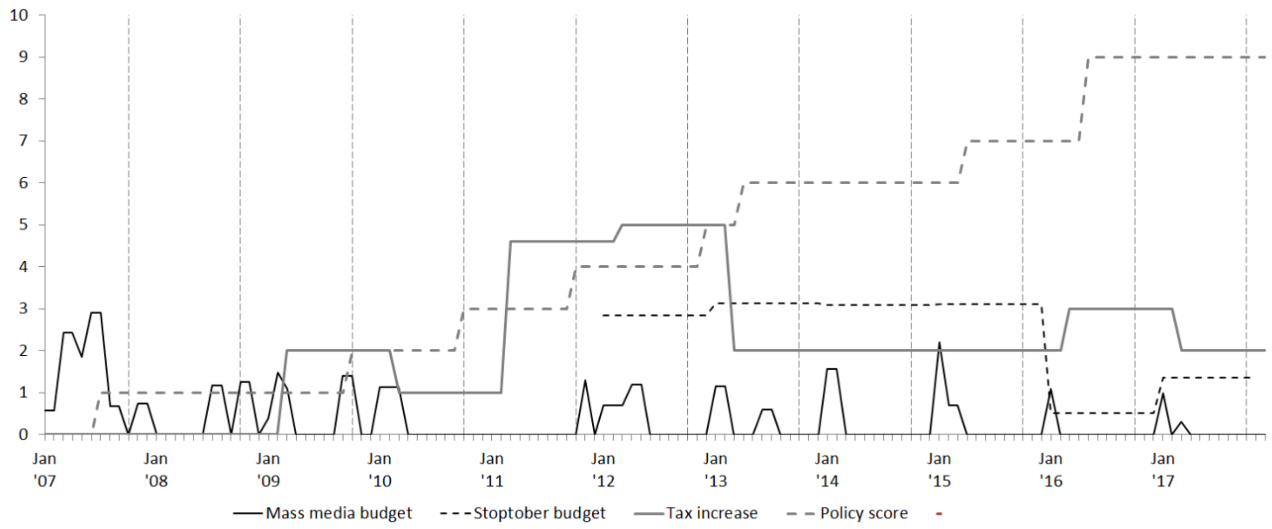


**Supplementary Figure 1:** 2007-2017 trends in total mass-media campaign budget in each month (in m£), Stoptober mass-media campaign budget per year (in m£), tax increase (in % above inflation) and cumulative tobacco control policy. Vertical lines indicate October months.
